# Roles and maturation of iron–sulfur proteins in plastids

**DOI:** 10.1007/s00775-018-1532-1

**Published:** 2018-01-18

**Authors:** Jonathan Przybyla-Toscano, Mélanie Roland, Frédéric Gaymard, Jérémy Couturier, Nicolas Rouhier

**Affiliations:** 1 Université de Lorraine, Interactions Arbres-Microorganismes, UMR1136, 54500 Vandoeuvre-lès-Nancy, France; 2 Biochimie et Physiologie Moléculaire des Plantes, CNRS/INRA/Université Montpellier 2, SupAgro Campus, 34060 Montpellier, France

**Keywords:** Biogenesis, Iron–sulfur proteins, Plastids, Electron transfer, Photosynthesis

## Abstract

One reason why iron is an essential element for most organisms is its presence in prosthetic groups such as hemes or iron–sulfur (Fe–S) clusters, which are notably required for electron transfer reactions. As an organelle with an intense metabolism in plants, chloroplast relies on many Fe–S proteins. This includes those present in the electron transfer chain which will be, in fact, essential for most other metabolic processes occurring in chloroplasts, e.g., carbon fixation, nitrogen and sulfur assimilation, pigment, amino acid, and vitamin biosynthetic pathways to cite only a few examples. The maturation of these Fe–S proteins requires a complex and specific machinery named SUF (sulfur mobilisation). The assembly process can be split in two major steps, (1) the de novo assembly on scaffold proteins which requires ATP, iron and sulfur atoms, electrons, and thus the concerted action of several proteins forming early acting assembly complexes, and (2) the transfer of the preformed Fe–S cluster to client proteins using a set of late-acting maturation factors. Similar machineries, having in common these basic principles, are present in the cytosol and in mitochondria. This review focuses on the currently known molecular details concerning the assembly and roles of Fe–S proteins in plastids.

## Introduction

Iron (Fe) and sulfur are critical elements for plant growth and development. Sulfur is notably required for cysteine and methionine synthesis, and is present in a large number of molecules, whereas Fe atoms are associated with many proteins as part of hemes, mono- or di-iron non-heme centers, or iron–sulfur (Fe–S) clusters. Chloroplasts and plastids in general, are highly demanding organelles for both elements due notably to the presence of a translation machinery and of the photosynthetic electron transfer chain. Besides, numerous metabolic pathways occurring totally or partially in this organelle are directly or indirectly dependent on the functioning of Fe–S proteins. This review is organized in three parts, describing how Fe and sulfur species get reduced and imported in chloroplasts, how the various types of Fe–S clusters are built from Fe and cysteine and incorporated into the tenths of client proteins, and finally which chloroplastic pathways/processes are dependent on these cofactors.

## Supply of iron and sulfur to plastids

### Iron transport

**Fig. 1:**
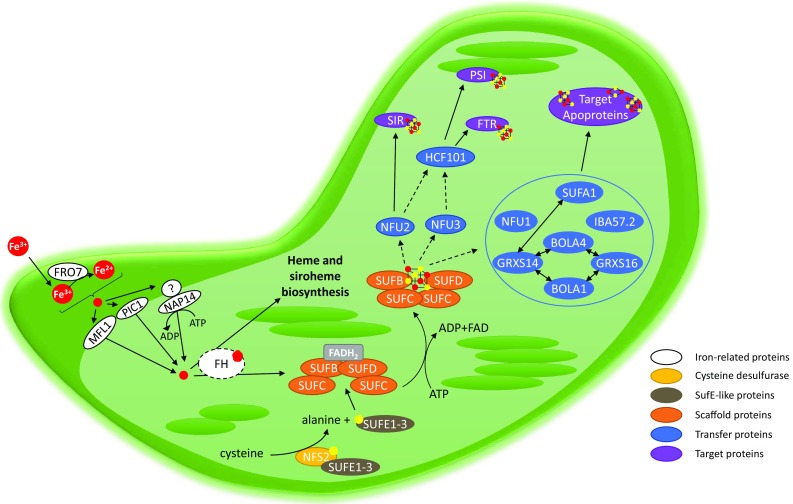
Working model for iron uptake and maturation of Fe–S proteins by the SUF machinery in plastids of eukaryotic photosynthetic organisms. Besides the putative Fe transporters located at the membrane of the chloroplast envelope, which would serve for providing the required Fe atoms to the SUF machinery, this scheme integrates the 17 putative SUF components. In the absence of stronger evidence concerning the implication of frataxin, it is not integrated among SUF components and is represented by a dashed circle. The color code associated with each protein function is indicated directly on the figure. The detailed description of the maturation process and the connections between the SUF proteins are described in the text. Except NFU2, NFU3, and HFC101, all maturation factors have been grouped in a blue circle in the absence of information concerning their precise function, but two-way arrows indicate that physical interactions have been observed between some proteins

Chloroplasts, where photosynthesis and heme synthesis occur, represent the major subcellular Fe sink in plant leaves [1]. Photosynthetic organisms uptake Fe from the soil using a sophisticated pumping system that differs between Poaceae and dicotyledon species, which developed, respectively, either a phytosiderophore-dependent chelation-based strategy or a reduction-based strategy (see [2] for an overview). In *Arabidopsis thaliana*, Fe is acquired in several steps. By extruding protons via the H^+^-ATPase AHA2 and coumarins via the PDR9 ABC transporter, *A. thaliana* can solubilize and chelate Fe^3+^ forms by lowering the soil pH. Then, the reduction of Fe^3+^ to Fe^2+^ is performed by the ferric reductase-oxidase (FRO) family protein, FRO2, before its uptake by the plasma membrane Fe transporter named iron-regulated transporter 1 (IRT1) [2]. In the cytosol of root cells, Fe complexes are formed with organic acids (malate or citrate) or nicotianamine before being translocated to the shoots and unloaded in the cytosol of mesophyll cells [3]. After this step, little is known concerning Fe acquisition by chloroplasts, its subsequent storage, and delivery to dedicated proteins and machineries. It is possible that a voltage-dependent transport system allows Fe^3+^-citrate complexes to pass the outer membrane of the plastid envelope [4]. Once in the chloroplastic intermembrane space, FRO7 may reduce ferric (Fe^3+^) to ferrous iron (Fe^2+^) via its reductase activity [5]. Several transporters located in the inner membrane of the chloroplast envelope are candidates for Fe import into the stroma (Fig. 1). The first one is named permease in chloroplast 1 (PIC1) [6]. Both knock-out and overexpression lines for this gene show abnormal chloroplast development and perturbed iron homeostasis and availability [6, 7]. The loss-of-function mutants are dwarf and chlorotic (even white), and they grow only heterotrophically. Moreover, they accumulate Fe into ferritins, the function of which is normally to protect this organelle from oxidative stress by sequestering Fe. The PIC1-overexpressing plants suffer from oxidative stress and leaf chlorosis likely due to a Fe overload in chloroplasts. Although this permease is mentioned to be part of the translocase of the outer/inner chloroplast membrane (Tic–Toc) complex in other studies, a Fe transport function is clear from the complementation of a yeast *fer3fer4* mutant which is defective in Fe uptake, leading to the conclusion that PIC1 may have a dual function [6]. Another putative Fe transporter, named NAP14 (non-intrinsic ABC protein 14), was identified from its homology with the ABC transporter FutC belonging to the FutABC iron uptake system in cyanobacteria [8]. As observed for *pic1*, a *nap14* knock-out mutant accumulates Fe in shoots, exhibits abnormal chloroplast structures, and shows deregulated levels of Fe homeostasis-related genes. However, in the absence of other Fut orthologs in *A. thaliana*, the question of whether NAP14 can work alone or in pair with other unidentified partners remains open. A third candidate transporter for Fe uptake in chloroplasts is mitoferrin-like1 (MFL1) [9]. However, although its gene expression is dependent on Fe supply and the protein is in principle located to the inner membrane of the chloroplast envelope, the growth of knock-out mutants is only moderately affected. While all these proteins seem to be involved in Fe homeostasis in chloroplasts, further characterization is urgently needed to clarify their exact function and respective importance.

### Sulfate import and reduction in plastids for the synthesis of cysteine, the sulfur donor of Fe–S clusters

Photosynthetic organisms use sulfate present in the soils as a primary source of sulfur. Sulfate is incorporated into the roots through an active proton/sulfate co-transport system located at the plasma membrane [10]. Once in the xylem, sulfate is transported to the shoots, unloaded into the cytosol of mesophyll cells, and then transported in the chloroplasts for its ATP-dependent reductive assimilation into sulfide (see [10] for review). The involved transporters all along these steps belong to the sulfate transporter (SULTR) family, which is composed of 12 members in *A. thaliana* that can be grouped into four classes. The SULTR3 class comprises the chloroplast-localized sulfate transporters [11]. The sulfide generated by the ferredoxin (FDX)-dependent sulfite reductase (SIR) will be used for cysteine biosynthesis by cysteine synthase, a complex of two enzymes, serine acetyltransferase (SAT) that uses acetyl-coA to form *O*-acetylserine (OAS) from serine and *O*-acetylserine-(thiol)-lyase (OAS-TL) which can substitute the acetyl moiety by sulfide to form cysteine. While the first steps of sulfate reduction into sulfide are clearly restricted to the chloroplasts, cysteine synthesis can also occur in the cytosol and in mitochondria owing to the ubiquitous expression of SAT and OAS-TL and exchange of sulfide across organelle membranes [10].

## The biogenesis of Fe–S proteins in chloroplasts by the SUF machinery

Several dozen of proteins containing Fe–S clusters are found in various subcellular compartments in the model plant *A. thaliana* as in other plants. Accordingly, in plant cells, three assembly machineries exist in plastids, in mitochondria, and in the cytosol, the latter being dedicated to the maturation of Fe–S proteins found both in the cytosol and in the nucleus. Whereas the chloroplastic sulfur mobilisation (SUF) machinery is autonomous, the cytosolic iron–sulfur assembly (CIA) machinery is dependent on the mitochondrial iron–sulfur cluster (ISC) machinery as it relies on a sulfur-containing compound generated in the first steps and exported from mitochondria by an ABC transporter. We invite the readers interested in the ISC and CIA machineries in plants to refer to the following recent reviews [12, 13]. For all these machineries and in particular the chloroplastic SUF machinery, the biosynthesis and delivery of Fe–S clusters can be separated in two major steps: their de novo assembly on scaffold proteins and their incorporation into final client proteins. This second step may necessitate the exchange and possibly conversion of Fe–S clusters between scaffold proteins and maturation factors including Fe–S cluster transfer proteins and targeting/recruiting factors. Repair mechanisms may eventually account for the recycling of damaged Fe–S clusters, which could be important in chloroplasts considering the presence of reactive oxygen and nitrogen species, but this will not be discussed further as information in plants is very scarce.

### The de novo Fe–S cluster assembly on scaffold protein

In chloroplasts, it seems now clear that the sole scaffold system is formed by the SUFBCD proteins (Fig. 1) [14]. The assembly of a Fe–S cluster on this scaffold complex theoretically requires the concerted action of several proteins as it requires the polypeptide backbones, ATP, Fe, and sulfur atoms and electrons. There are still many uncertainties about the involved actors in plants and the molecular details. Thus, we will often make analogies to the *Escherichia coli* SUF system, which has been better characterized. The best, not to say the only, well-characterized actors in plants of this assembly complex are proteins required for the production and transfer of the required sulfur. The NFS2 protein (formerly referred to as CpNifS) is a pyridoxal-l-phosphate (PLP)-dependent cysteine desulfurase, which catalyzes the extraction of the sulfur atoms from cysteine, producing a persulfide group on a catalytic cysteine with the concomitant release of an alanine (Fig. 1) [15]. As a class II cysteine desulfurase, similar to the bacterial SufS orthologs, the accessibility of the persulfide group is limited by the presence of a β-hairpin near the catalytic cysteine [16, 17]. For this reason, the transfer of sulfur atoms to the scaffold complex relies on an additional protein named SUFE. In *A. thaliana*, there are three SUFE proteins (SUFE1-3) targeted to chloroplasts [18]. In addition to the SUFE domain, SUFE1 has a C-terminal BOLA domain, the role of which is unknown but may prefigure a control by glutaredoxins (GRXs, see below) and SUFE3 possesses a quinolinate synthase (NadA) domain at the C-terminus, which is involved in NAD biosynthesis [18, 19]. As shown for the corresponding *E. coli* couple [20], each SUFE protein enhances the cysteine desulfurase activity of NFS2 by accepting the persulfide group on its own catalytic cysteine, thus serving as a relay to the scaffold system [18, 19]. At the structural level, *A. thaliana* NFS2 is a dimeric protein with two distant active sites, which suggests that the functional NFS2-SUFE unit should be a heterotetramer [17]. In addition to the existence of additional domains in SUFE1 and SUFE3, the existence of three SUFE isoforms may be also linked to their expression pattern as for instance *SUFE2* is mostly expressed in flowers [18]. The central role of these proteins has been validated by genetic studies, since the study of knock-out *A. thaliana* lines proved that *NFS2*, *SUFE1*, *SUFE3,* and *SUFBCD* genes are essential [14, 18, 21, 22]. The use of RNAi lines showed that NFS2 and SUFBCD are required for the maturation of all plastidial Fe–S proteins tested so far [14, 22].

In *E. coli* as in *A. thaliana*, the scaffold complex is probably composed by three subunits, SUFB, SUFC, and SUFD, very likely in a 1:2:1 stoichiometry and will be referred to as SUFBC_2_D (Fig. 1) [14, 20]. It seems that NFS2, SUFEs, and SUFBCD do not form a large and stable complex as recently shown in the case of the mitochondrial ISC system in yeast and human [23, 24]. Indeed, some in vitro biochemical analyses using the bacterial SufS, SufE, and SufBCD enzymes indicated that SufS does not seem to make stable interactions with SufBCD, unlike SufE whose presence is absolutely required for an efficient Fe–S cluster reconstitution in vitro on SufBCD [20, 25]. Besides, it has been shown that the presence of SufC, but not SufD, is required for the transfer of the sulfur atoms bound to *E. coli* SufE to SufB. Upon ATP binding, the SufC ATPase would induce structural changes on SufB and SufD that are necessary for Fe–S cluster binding [26]. Some residues important for these interactions have been identified from the 3D structures and validated by mutagenesis [26, 27]. Among the numerous cysteines present in SufB, the primary sulfur acceptor would be the conserved Cys254 (*E. coli* numbering). This sulfur atom would then be transferred to Cys405, one of the Fe–S cluster ligands owing to the existence of a tunnel inside the β-helix core domain of SufB [27]. The question of which type of Fe–S clusters is bound to this complex has been investigated in detail. It was shown that *E. coli* SufB alone can assemble both [Fe_2_S_2_] and [Fe_4_S_4_] clusters in vitro and that a conversion from the [Fe_4_S_4_]-loaded SufB form to a stable [Fe_2_S_2_]-loaded form is possible upon exposure to air [25, 28]. However, based on the structure of an apoSufBCD complex, it was proposed that a histidine of SufD may be a Fe–S cluster ligand [26]. Consistently, a mutated variant for this histidine lost the ability to assemble a Fe–S cluster in vivo, and both SufC and SufD were required for the in vivo maturation of SufB [29]. In this cellular context, *E. coli* SufBC_2_D complex mostly binds a [Fe_4_S_4_] cluster with some residual amount of linear [Fe_3_S_4_] clusters [29]. Hence, it is very likely that the SufBC_2_D scaffold binds a [Fe_4_S_4_] cluster in vivo and considering the conservation between *A. thaliana* and *E. coli* sequences, we anticipate that this mechanism should also prevail for plant proteins. However, we cannot completely rule out that SufBC_2_D or other forms, such as the SufB_2_C_2_ form detected with *E. coli* proteins [29], can bind other cluster types in some conditions. For instance, transcriptomic data indicate that the *SUFB*, *SUFC,* and *SUFD* genes may not be co-expressed in all organs and cell types of *A. thaliana*.

At this stage of the assembly process, there are many other crucial questions concerning the source of electrons required for the reduction of Fe^3+^ to Fe^2+^ or of the persulfide (S^0^) to a sulfide (S^2−^), the source of Fe, and the control of its entry in the complex. In this respect, it is important to note that the SufBC_2_D complex was purified with a bound reduced flavin-adenine dinucleotide (FADH_2_) molecule [29, 30]. While SufB alone can bind the flavin in vitro [30], SufD is also required in vivo [29]. It is currently believed that this FADH_2_ provides the necessary reducing equivalents for the reduction of ferric iron. Since FAD is released from the complex upon oxidation, an external regeneration system is needed, which could be possibly an FDX or an NADPH-dependent flavin reductase.

The mechanisms and actors involved in the delivery of Fe for Fe–S cluster biosynthesis in plastids are completely unknown. The Fe–Storage proteins, ferritins, have been excluded from Fe donor candidates, because an *Arabidopsis* mutant (*fer1*-*3*-*4*) for the three ferritins found in leaves has no apparent phenotype [31], while mutant plants modified for the expression of these early biogenesis factors are either lethal or at least strongly affected. Another candidate for Fe delivery is a small acidic protein with iron-binding properties named frataxin. In the mitochondrial ISC machinery, frataxin controls iron entry in the assembly complex by activating sulfide formation by the cysteine desulfurase [32, 33]. In this complex, frataxin can interact both with the cysteine desulfurase and the ISCU scaffold protein. Except for a few organisms like *Z. mays*, there is usually a single gene coding for frataxin (FH) in plants. While frataxin was believed for a long time to be exclusively located in mitochondria, it was recently reported that *A. thaliana* FH (AtFH) and two isoforms from *Z. mays* may have a dual targeting into both mitochondria and plastids [34, 35]. According to this possible chloroplastic localization*, Arabidopsis FH*-deficient plants show a decrease in the heme content [36]. Moreover, they present a decrease in the total chlorophyll content, in the levels of two plastidial FDXs and in nitrite reductase (NIR, a siroheme-containing enzyme) activity which could explain the observed changes in the rate of the photosynthetic electron transport chain [35]. The impact on heme content would be in good agreement with the described interaction between yeast frataxin and ferrochelatase, the terminal enzyme of heme synthesis performing porphyrin metalation [37]. All these observations suggest an impairment of the plastidial Fe–S cluster biosynthesis and/or of the heme or siroheme biosynthesis, although stronger and more direct biochemical evidence is still required.

### Delivery and trafficking of preformed Fe–S clusters by maturation factors

The preformed Fe–S cluster on the SUFBC_2_D complex, be it a [Fe_2_S_2_] or a [Fe_4_S_4_] cluster, has then to be correctly targeted to client apoproteins. This requires several other proteins referred to as maturation factors. Among these, one could differentiate the so-called Fe–S cluster transfer/carrier proteins (belonging to NFU, SUFA, GRX, and HCF101 families) from targeting factors (belonging to BOLA and IBA57 families) which, contrary to the proteins of the first group, are not able to bind Fe–S clusters by themselves, although BOLAs do it in complex with GRXs [38, 39]. It is interesting to note that all proteins of these families have mitochondrial counterparts in the ISC machinery, whereas the components forming the eukaryote-specific CIA machinery usually belong to different protein families [12]. This analogy to the mitochondrial system is the reason why some of these plastidial members, whose role in the maturation of Fe–S proteins in plastid has not been yet established, have been included in this section. The current model for these steps in the plant mitochondrial ISC machinery derives mainly from studies conducted in yeast and human and can be summarized as follows [40]. A glutaredoxin (GRXS15 in plants) is the primary transfer protein receiving a [Fe_2_S_2_] cluster from ISCU proteins. This cluster can be either directly inserted into [Fe_2_S_2_]-recipient apoproteins or used to build [Fe_4_S_4_] clusters on a heterocomplex formed by ISCAs and possibly IBA57. Some mechanistic and structural aspects of the cluster conversion from the [Fe_2_S_2_]-loaded GLRX5 form to the [Fe_4_S_4_]-loaded ISCA1-2 form have been recently delineated using human proteins [41, 42]. Then, the insertion of the [Fe_4_S_4_] clusters into client Fe–S proteins might be direct or facilitated by NFU and BOLA proteins that likely act in concert for the maturation of specific targets notably the lipoate synthase or by IND1/INDH, a close HCF101 homolog, which seems specific for the respiratory chain complex I.

The current genetic and biochemical evidence indicate that this sequence of events should be very different for the plastidial SUF machinery (Fig. 1). Although the two plastidial isoforms, named GRXS14 and GRXS16, have the ability to bind the regular [Fe_2_S_2_] cluster in homodimer (or in heterodimer with BOLA, see below) and to complement a yeast mutant for the mitochondrial Grx5 [43], strong genetic and physiological evidence for a similar involvement in plants is still missing. Single mutants for each of these genes have no phenotype when grown under standard conditions, whereas plants overexpressing GRXS14 have a decreased chlorophyll content [44]. Considering that several enzymes involved in chlorophyll catabolism require Fe–S clusters, this may constitute a first hint towards a role of GRXS14 in the maturation of specific client proteins in this pathway. Counterintuitive to this first observation, plants lacking GRXS14 showed accelerated chlorophyll loss compared to wild-type plants when exposed to prolonged darkness, suggesting more complex connections [44]. A redundancy may exist between both plastidial GRXs, since a double mutant with about 20% GRXS16 remaining exhibits a 20% biomass reduction in standard conditions compared to wild-type plants. However, this phenotype is not exacerbated under stress conditions. Overall, unlike the knock-out mutant of mitochondrial GRXS15 which is embryo-lethal [45], these results point either to non-essential roles of these isoforms or to a redundant function with the remaining GRXS16 level being sufficient to sustain an essential role similar to GRXS15.

Concerning BOLA proteins, several roles have been proposed, but only those connected to their participation in Fe metabolism have been really validated [46]. Their involvement in Fe–S cluster biogenesis was demonstrated from the study of *bol1/3* mutant in yeast and of human patients defective for the mitochondrial BOLA3. Both types of cells display protein lipoylation defects due to the incorrect maturation of lipoate synthase and a decrease in activity for some other [Fe_4_S_4_] proteins as aconitase and succinate dehydrogenase [47–49], whereas human patients also have defects in the mitochondrial respiratory complexes I and III [49]. Three isoforms with a BOLA domain are found in plant chloroplasts. As already mentioned, the C-terminal region of SUFE1 contains a BOLA domain. The two other isoforms, BOLA1 and BOLA4, comprise a single domain. Both BOLA4 and SUFE1 could also be targeted to mitochondria [21, 50]. Interactions between these plastidial BOLA proteins and GRXS14 and GRXS16 have been demonstrated both in vitro and in planta [38, 50]. These proteins can in fact form both apo-heterodimers and holo-heterodimers bridging a [Fe_2_S_2_] cluster [39], as also demonstrated for bacterial, yeast, and mammalian isoforms [46]. In this respect, it is interesting to note that adding BOLA to a GRX homodimer converts it to a more stable holo GRX-BOLA heterodimer. This interconversion might represent a regulatory mechanism either to shut down or activate some specific pathways by favouring one target over another. At the structural level, all BOLA isoforms have a similar well-conserved fold [39]. Two subgroups can, however, be distinguished based on the length of the β1–β2 loop referred to as the variable [C/H] loop, because it contains one of the ligands provided by BOLA either a cysteine or a histidine, the second ligand being a totally conserved histidine found in the α3–β3 loop [39, 48]. Other cysteine ligands are provided by a glutathione molecule and by the one present in the conserved CGFS signature of the GRX partner, as in regular GRX homodimers [51]. While there is no true ortholog of yeast Bol3 in plants, the observation that Bol3 might interact with Nfu1 rather than with Grx5 in yeast could point to a different role in the late steps of the mitochondrial system [47, 48]. Although single *bol1* and *bol3* mutants do not have phenotypes and the respective molecular roles of Bol1 and Bol3 are still unclear, a connection between Bol3 and Nfu1 is also evident from the quite similar phenotype of the *bol1/3* and *nfu1* mutant cells [47].

In mitochondria, ISCA proteins are central for the maturation of [Fe_4_S_4_] proteins, presumably ensuring the conversion of [Fe_2_S_2_] centers into [Fe_4_S_4_] centers. In bacteria, the different A-type isoforms (IscA, SufA, ErpA) are also required for the maturation of [Fe_4_S_4_] proteins, even though in vitro studies demonstrated that *Azotobacter vinelandii* IscA, for example, can reversibly cycle between [Fe_2_S_2_] and [Fe_4_S_4_] forms through electron reductive coupling or oxidative cleavage [52]. Some biochemical redundancy seems to exist between them as demonstrated for the Fe–S cluster assembly of IspG and IspH, two enzymes involved in isoprenoid synthesis and also present in plant chloroplasts [53, 54]. In plastids, the only representative of this family should be SUFA1, also referred previously to as CpISCA and ISCA-I [55, 56]. As an A-type carrier protein, SUFA1 possesses the three characteristic conserved cysteines [54, 55] that allow the binding of a [Fe_2_S_2_] center in a dimer as observed upon in vitro Fe–S cluster reconstitution assays [55–57]. According to the ISC model, Fe–S cluster transfer experiments showed that GRXS14 can efficiently and unidirectionally transfer its [Fe_2_S_2_] cluster to SUFA1; however, there was no sign of a [Fe_4_S_4_] cluster formation [57]. Using recombinant proteins, it was shown in vitro that an apo-SufA from *E. coli* could promote the maturation of an apo-FDX from a [Fe_4_S_4_]-loaded SufBC_2_D scaffold, indicating that SUFA proteins would directly interact with the scaffold but also that it facilitates Fe–S cluster conversion. Nevertheless, knock-out mutants have no visible phenotype when grown under standard conditions, indicating that the role of SUFA1 is dispensable [55, 56]. Whether it is involved in the maturation of [Fe_2_S_2_] proteins, [Fe_4_S_4_] proteins or both remains thus to be determined.

It is getting clear that, in yeast and human mitochondria, ISCA proteins interact with IBA57 (Iron–Sulphur cluster assembly factor for Biotin synthase- and Aconitase-like mitochondrial proteins with a mass of 57 kDa). They form a complex involved in the maturation of several [Fe_4_S_4_] proteins including radical-*S*-adenosylmethionine (SAM) proteins, homoaconitase, aconitase, biotin synthase, and lipoic acid synthase [58, 59]. Depletion of the *E. coli* ortholog YgfZ also affects some [Fe_4_S_4_] proteins such as succinate dehydrogenase, fumarase, dimethylsulfoxide reductase, and MiaB, an enzyme involved in tRNA thiolation [58–60]. The two orthologs found in *A. thaliana*, IBA57.1 and IBA57.2, are, respectively, localized in mitochondria and plastids [61]. It is interesting to note that both isoforms can complement the growth defects of an *E. coli ygfZ* mutant observed on a minimal medium or upon oxidative stress [60]. This is the only physiological information obtained so far for these plant isoforms, since an *Arabidopsis iba57.1* mutant is embryo-lethal and an *iba57.2* mutant has not been described. While the exact function of IBA57 is still unknown, it is important to note that there is a conserved cysteine residue in a KGCY-x-GQE-x3-R/K motif, which is almost the only conserved motif in this protein family [62]. Moreover, consistent with the structural similarity of IBA57 with folate-dependent enzymes [63], *E. coli* YgfZ can bind tetrahydrofolate [60].

**Fig. 2:**
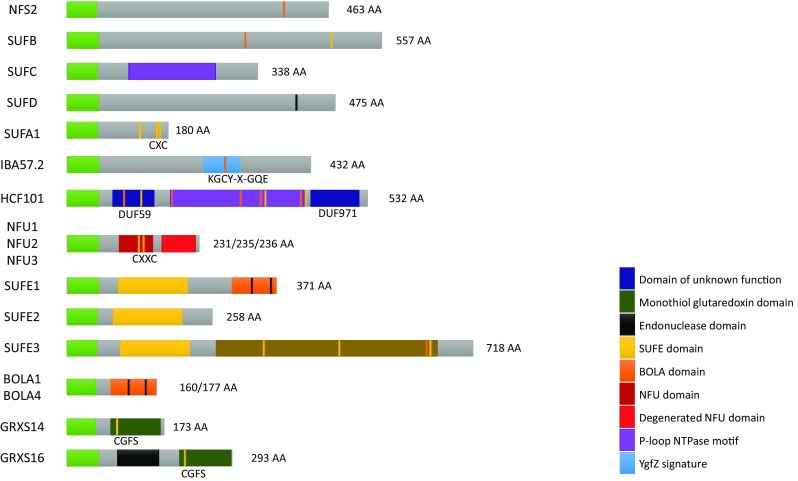
Protein domain organization of SUF components. The domains (identified using pfam or the NCBI conserved domain tools) present in SUF components have been represented using the color code defined on the figure. Except for the chloroplastic targeting sequence (light green boxes), the domains are represented at scale, with the length in amino acids of the *Arabidopsis* proteins indicated. The Fe–S binding cysteine and histidine residues are represented in yellow and black, respectively, while other conserved cysteines are in orange, although their function is sometimes unclear if any

Another category of proteins strictly required for the maturation of [Fe_4_S_4_] clusters is the NFU family that exists in all kingdoms. In mitochondria, the study of the yeast mutant and several human patients indicates that NFU1 is required for the maturation of lipoate synthase, which affects several ketoacid dehydrogenases dependent on lipoic acid, and for the maturation of complexes I, II or III depending on the patients [49, 64]. *A. thaliana* encodes five NFU isoforms, two (NFU4 and NFU5) should be targeted to mitochondria, and three (NFU1, NFU2, and NFU3) are localized in chloroplasts [65, 66]. All these proteins share an NFU domain possessing a CXXC motif necessary for the binding of a [Fe_4_S_4_] in a dimer [67]. Chloroplastic isoforms have an additional NFU domain in the C-terminal extremity which does not have the cysteine residues, whereas mitochondrial isoforms have an additional N-terminal domain of unknown function (Fig. 2) [65, 68]. Loss-of-function *nfu2* and *nfu3* mutants have a dwarf phenotype with pale green leaves [69, 70]. By coupling chlorophyll fluorescence and P700 absorption measurements to western blot analyses, it was shown that this phenotype is due to the impairment of photosystem I (PSI) architecture and activity which is explained by a defect in the maturation of the three [Fe_4_S_4_] clusters assembled in the psaA, psaB, and psaC subunits. The only other notable and robust molecular default observed is that the SIR level and activity are decreased in *nfu2* [14, 65, 70, 71]. The fact that a double *nfu2*-*nfu3* mutant is lethal [69] indicates that both NFU isoforms should have partially overlapping functions. This raises also the question of their contribution relatively to the high chlorophyll fluorescence 101 (HCF101) protein, a plastidial 51 kDa protein belonging to the NTPase protein family (Fig. 2). The *hcf101 Arabidopsis* mutant plants have globally similar molecular defects, although this is exacerbated as the strongest allele is lethal at the seedling stage and the decrease in the amounts of PSI subunits is stronger, almost complete [72–74]. Besides, there is a decrease in the ferredoxin-thioredoxin reductase (FTR) levels, another [Fe_4_S_4_] protein [72]. Overall, in accordance with the capacity of *Arabidopsis* NFU2 and HCF101 to bind [Fe_4_S_4_] cluster in vitro [67, 73], this indicates that all these proteins are required for the maturation of [Fe_4_S_4_] proteins, particularly PSI subunits, and that HCF101 would act downstream of NFU2 and NFU3 (Fig. 1).

In summary, there are currently ten putative maturation factors in the SUF machinery for several dozens of plastidial client proteins. The role of some of these maturation factors still awaits confirmation not to speak about their connections and hierarchical organization. There is also an urgent need to learn more about how specificity towards target proteins is achieved and about the molecular and structural aspects of these interactions.

## Functional diversity among client Fe–S proteins in plastids

### Fe–S clusters in the functioning and protection of the photosynthetic electron transport chain

Among other functions, Fe–S clusters have a crucial role in electron transfer reactions, and thus, several Fe–S proteins are found in the thylakoid membrane as part of the photosynthetic electron transport chain. A Rieske-type Fe–S cluster, i.e., a [Fe_2_S_2_] cluster ligated by two cysteines and two histidines, is found in the Rieske protein of the cytochrome b_6_f complex. In the genome of eukaryotes and in some cyanobacteria, the Rieske protein is encoded by a single gene named photosynthetic electron transfer C (*petC*), whereas in most cyanobacteria, there are additional isoforms whose physiological function is still uncertain [75]. The absence of the petC proteins is lethal in the early developmental stages both in *A. thaliana* and cyanobacteria (Table 1) [75, 76]. Three low potential [Fe_4_S_4_] clusters are attached to the thylakoid membrane but face the reducing, stromal side of PSI, and function in series. The first, referred to as F_X_, is associated with a PsaA–PsaB heterodimer via cysteine residues, while the two others, named F_A_ and F_B_, are bound to PsaC [77]. These clusters transfer electrons to FDXs, small soluble proteins, which contain a classical [Fe_2_S_2_] cluster, e.g., rhombic cluster ligated by four cysteines. The nuclear genome of algae and plants harbours a variable number of *FDX* homologs, differentially expressed in plant organs or at different development stages or in response to different stimuli [78]. The *A. thaliana* genome contains four genes encoding four well-described plastidial *FDXs* (*Fd1* to *Fd4*) and at least two additional genes, referred to as *FdC1* and *FdC2*, encoding proteins bearing C-terminal extensions and whose functions remain elusive [79, 80]. In specific physiological situations such as environmental constraints, FDXs can recycle electrons to the plastoquinone pool, contributing to the so-called cyclic electron flow [81]. The major cyclic pathway is dependent on the PGR5 (proton gradient regulation 5)/PGRL1 (PGR5-like photosynthetic phenotype 1) proteins [82]. The other involves the NAD(P)H dehydrogenase (NDH) complex. In higher plants, it forms a large complex associated with PSI, which is composed of 11 plastid-encoded subunits, some additional nuclear-encoded subunits, and auxiliary factors [83]. Among these, the NDH-I and NDH-K subunits bind two and one [Fe_4_S_4_] clusters, respectively [84]. While *Arabidopsis* knock-out mutants for *NDH*-*I* and *NDH*-*K* genes have not been characterized, tobacco knock-out mutants of *ndh* genes usually have no phenotype under standard conditions but are sensitive to environmental stresses [82].

**Table 1:** Phenotypes of *A. thaliana* mutant lines for plastidial Fe–S proteins

Short Name	AGI number	Cluster type	Type of mutants	Mutant phenotypes	References
DHAD	At3g23940	[Fe_2_S_2_]	Knock-out	Embryo-lethal	Zhang et al. [149]
			Knock-down	Shorter root, hypersensitive to salt stress	
IPMI (LSU1)	At4g13430	[Fe_4_S_4_]	Knock-down	Pleiotropic growth abnormalities	Sureshkumar et al. [152], Knill et al. [151]
DWARF27.1	At1g03055	[Fe_4_S_4_]	Knock-down	Increase in axillary rosette branches	Waters et al. [168]
DWARF27.2	At1g64680	[Fe_4_S_4_]	Not yet described		
DWARF27.3	At4g01995	[Fe_4_S_4_]	Not yet described		
ISPG	At5g60600	[Fe_4_S_4_]	Knock-out	Albino phenotype, proplastid growth and thylakoid membrane formation affected	Gutiérrez-Nava et al. [163]
ISPH	At4g34350	[Fe_4_S_4_]	Knock-out	Albino phenotype, proplastid growth and thylakoid membrane formation affected	Gutiérrez-Nava et al. [163], Hsieh and Hsieh [165], Guevara-García et al. [164]
THIC	At2g29630	2x [Fe_4_S_4_]	Knock-down	Lethal (development arrested at the cotyledon stage with chlorotic phenotype)	Raschke et al. [140], Kong et al. [142]
NIR	At2g15620	[Fe_4_S_4_], siroheme	X-ray mutagenesis	Lethal in barley unless a nitrogen source is provided	Duncanson et al. [120]
SIR	At5g04590	[Fe_4_S_4_], siroheme	Knock-out	Lethal	Khan et al. [119]
			Knock-down	Early seedling lethal	Khan et al. [119]
SIRB	At1g50170	[Fe_2_S_2_]	Knock-out	Seedling lethal (post-germination arrest)	Saha et al. [126]
ATase1	At2g16570	[Fe_4_S_4_]	Knock-out	No phenotype	Hung et al. [144]
ATase2	At4g34740	[Fe_4_S_4_]	X-ray mutagenesis	Small and albino/pale reticulated leaves, cell division affected	Kinsman and Pyke [171], Hung et al. [144], van den Graaf et al. [146], Rosar et al. [172]
ATase3	At4g38880	[Fe_4_S_4_]	Not yet described		
APR1	At4g04610	[Fe_4_S_4_]	Not yet described		
APR2	At1g62180	[Fe_4_S_4_]	Knock-out	None but increased sensitivity to selenate tolerance	Grant et al. [173]
APR3	At4g21990	[Fe_4_S_4_]	Not yet described		
cLIP1	At5g08415	2x [Fe_4_S_4_]	Not yet described		
GLT1	At5g53460	[Fe_3_S_4_]	Knock-out	No phenotype but decreased chlorophyll content, growth defect under low CO_2_	Lancien et al. [123]
GLU1	At5g04140	[Fe_3_S_4_]	Knock-down	Dwarf photorespiratory phenotype	Somerville and Ogren [125], Coschigano et al. [122], Lancien et al. [123]
GLU2	At2g41220	[Fe_3_S_4_]	Knock-down	No phenotype	Potel et al. [124]
DjC17	At5g23240	[Fe_4_S_4_]	Knock-out	Defective root hairs	Petti et al. [91]
DjC18	At2g42750	[Fe_4_S_4_]	Not yet described		
ndhI	AtCg01090	2x [Fe_4_S_4_]	Not yet described		
ndhK	AtCg00430	[Fe_4_S_4_]	Not yet described		
petC	At4g03280	rieske [Fe_2_S_2_]	Knock-out	Seedling lethal	Maiwald et al. [76]
psaA	AtCg00350	[Fe_4_S_4_] with psaB	Not yet described		
psaB	AtCg00340	[Fe_4_S_4_] with psaA	Not yet described		
psaC	AtCg01060	2x [Fe_4_S_4_]	Not yet described		
TIC55	At2g24820	rieske [Fe_2_S_2_]	Knock-out	No phenotype	Boij et al. [106], Hauenstein et al. [104]
			Knock-down	No phenotype	Tanaka et al. [174]
PAO (ACD1)	At3g44880	rieske [Fe_2_S_2_]	Knock-out	Age- and light-dependent cell death phenotype in leaves and flowers. Stay-green phenotype in the dark	Pružinská et al. [114]
		rieske [Fe_2_S_2_]	Knock-down	Light-dependent lesion mimic phenotype, increased sensitivity to biotic and mechanic stresses	Greenberg and Ausubel [112], Yang et al. [175]
PTC52 (ACD1-like)	At4g25650	rieske [Fe_2_S_2_]	Knock-out	No phenotype	Boij et al. [106]
CMO	At4g29890	rieske [Fe_2_S_2_]	Not yet described		
CAO	At1g44446	rieske [Fe_2_S_2_]	X-ray mutagenesis	Pale green phenotype with no Chl b, highly photosensitive	Espineda et al. [103], Ramel et al. [107]
HCAR	At1g04620	2x [Fe_4_S_4_]	Knock-out	No phenotype, stay-green mutant upon dark exposure	Meguro et al. [108]
NEET	At5g51720	Neet-[Fe_2_S_2_]	Knock-down	Late bolting, early senescence	Nechushtai et al. [170]
SUFE3	At5g50210	[Fe_4_S_4_]	Knock-out	Lethal	Murthy et al. [18]
Fd1	At1g10960	[Fe_2_S_2_]	Knock-down	Enhanced linear electron flow	Hanke and Hase [72]
Fd2	At1g60950	[Fe_2_S_2_]	Knock-out	Growth arrest and inactivation of photosynthesis	Voss et al. [176]
			Knock-down	Lower biomass accumulation and retarded linear electron flow	Hanke and Hase [78]
Fd3	At2g27510	[Fe_2_S_2_]	Knock-down	Photoinhibition, with a reduction in maximum PSII yield following dark adaptation	Hanke and Hase [78]
Fd4	At5g10000	[Fe_2_S_2_]	Not yet described		
FdC1	At1g32550	[Fe_2_S_2_]	Not yet described		
FdC2	At4g14890	[Fe_2_S_2_]	EMS mutagenesis	Yellow–green leaf phenotype in rice	Li et al. [177]; Zhao et al. [178]
FTR	At2g04700	[Fe_4_S_4_]	Knock-out	Lethal	Wang et al. [97]
			Virus-induced silencing	Chlorosis, abnormal chloroplast development	Wang et al. [97]

All described phenotypes come from studies performed with *A. thaliana* unless otherwise stated in the “mutant phenotype” column

In eukaryotic microalgae and cyanobacteria, an additional pathway directly coupled to the photosynthetic electron transport chain and involving hydrogenases allows the photoproduction of ATP at the expense of reductant synthesis in specific conditions such as the response to anaerobiosis or anoxia. *Chlamydomonas reinhardtii* contains two [FeFe]-hydrogenases, namely HYDA1 and HYDA2, which will produce molecular hydrogen H_2_ from protons by accepting electrons from FDXs. These HYDA contain a complex Fe–S cluster at their active sites, the H-cluster that is essential for catalytic activity [85]. It consists of a classic [Fe_4_S_4_] cluster linked to a complex 2Fe sub-cluster [86]. Whereas the [Fe_4_S_4_] cluster is assembled by the regular SUF machinery, the sub-cluster requires specific maturation proteins, HYDE, HYDF, and HYDG, for this assembly. The HYDE and HYDG gene products are radical-SAM enzymes, whereas HYDF is a P-loop NTPase protein constituting a scaffold assembly platform. These proteins incorporate themselves [Fe_4_S_4_] clusters that are required for their activity.

As oxygenic photosynthesis releases massive amounts of oxygen from water, reactive oxygen species are routinely generated and damage some proteins in many physiological conditions. Thus, several proteins are implicated in the repair and protection of the photosystems and their antennae. One of these, photosystem II protein33 (PSB33), is an integral membrane protein, which contributes to the maintenance of PSII-light-harvesting complex II (LHCII) supercomplex organization in response to changing light levels [87]. Whereas the *Arabidopsis* protein is annotated as containing a Rieske-type Fe–S cluster by analogy to some bacterial counterparts, it does not have the Fe–S binding residues contrary to *C. reinhardtii* ortholog. Other *Chlamydomonas* proteins, referred to as CDJ3-5 for chloroplast-targeted DnaJ-like proteins, might be important for PSII protection. It was shown that CDJ3 and CDJ4 which interact with chloroplast ATP-bound HSP70B (heat-shock protein 70B) and are located either in the stroma or attached to thylakoids, respectively, are able to bind a [Fe_4_S_4_] cluster [88]. Based on the fact that HSP70B plays a role in the repair and protection of PSII (Photosystem II) from photoinhibition [89], and together with the CDJ2 paralog in the biogenesis/maintenance of thylakoid membranes [90], we could speculate that CDJ3-5 may have a similar role. However, this has not been addressed so far for *Arabidopsis* orthologs, DjC17 and DjC18. There is no biochemical information on these proteins and genetic evidence has been obtained only for DjC17, the mutation of which results in an altered root hair development and reduced hair length due to aberrant cortical cell division [91].

### A multitude of ferredoxin-dependent Fe–S proteins and pathways

#### The formation of reducing equivalents

**Fig. 3:**
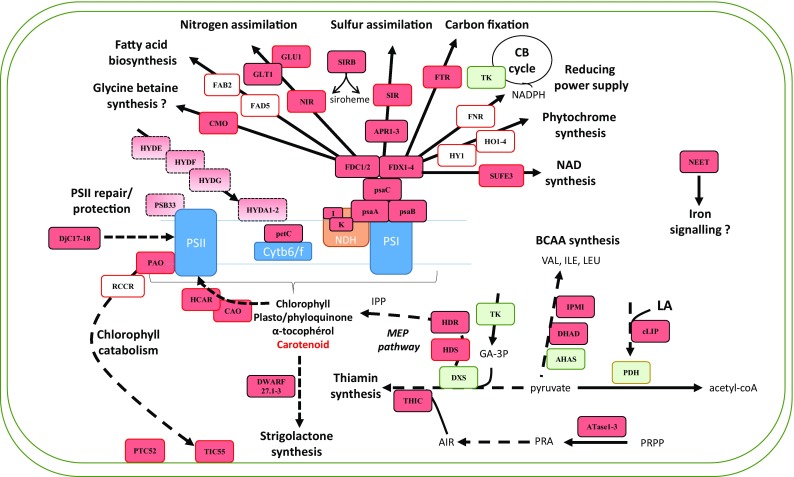
Fe–S protein-dependent metabolic processes in plastids. Fe–S proteins are represented by dark red boxes. The light red boxes indicate specificities found in algae either, because they do not exist in terrestrial plants or in the case of PSB33, because only the algal isoforms should incorporate a Fe–S cluster. Known FDX-dependent enzymes have a red outline. Enzymes in green or outlined in orange use, respectively, thiamin or lipoic acid as cofactors. Note that PDH is dependent on both cofactors. For APR, DWARF, ATase, FDX, FDC, DjC, and HYDA1/2, there are several close isoforms which have not been distinguished. The nomenclature used is the one of *A. thaliana* except for algal enzymes whose name is from *C. reinhardtii*. Abbreviations for all enzyme names can be found in the text. Other abbreviations are: *LA* lipoic acid, *BCAA* branched-chain amino acids, *IPP* isopentenyl diphosphate, *GA-3P* glyceraldehyde 3-phosphate, *AIR* 5-aminoimidazole ribonucleotide, *PRA* phospho-ribosylamine, *PRPP* 5-phosphoribosyl-1-pyrophosphate

FDXs are soluble proteins positioned at a metabolic crossroad, controlling the electron flow necessary for CO_2_ fixation, nitrogen, and sulfur assimilation but also chlorophyll metabolism to cite a few examples (Fig. 3). Their primary role is to transfer electrons to various acceptors in the stroma, in the thylakoids, and in the inner membrane, including a large variety of Fe–S proteins but also proteins containing heme and non-heme iron centers and flavoproteins [92, 93]. Among the latter category, ferredoxin-NADP reductase (FNR) may be the most important one, since it will drive most of these electrons for the regeneration of NAPDH, which will then supply in particular the Calvin–Benson cycle. It is worth nothing that a significant fraction of FNR is bound to the thylakoid membrane and it could participate to the cyclic electron flow via the PGR5-dependent pathway by interacting with PGLR1 and recruiting FDXs. Another enzyme crucial for carbon fixation and metabolism, in general, is the FTR. This key enzyme, which is almost uniquely found in photosynthetic organisms, catalyzes the reduction of most thioredoxins (TRXs) found in plastids, thus indirectly participating to the regulation of all TRX-dependent targets in a light-dependent manner [94]. FTR is a heterodimer composed of a catalytic and a variable subunit [95]. In *A. thaliana*, there is a single gene for the catalytic subunit (FTRB) but two for the variable subunits (FTRA1 and FTRA2). The function of the [Fe_4_S_4_] cluster found in the catalytic subunit is to aid for the reduction of a redox-active disulfide, which reduces, in turn, the TRX disulfide [96]. Given the numerous functions played by plastidial TRXs, it is not surprising that an *ftr* knock-out mutant for the catalytic subunit is lethal. However, a virus-induced gene silencing (VIGS) approach led to plants exhibiting a sectored chlorotic leaf phenotype [97]. It could be observed that these plants have (1) an abnormal chloroplast biogenesis, (2) a reduced photosynthetic performance as measured by the photochemical activities, the amount of assembled photosystems and CO_2_ assimilation rates, and (3) a defective PEP (plastid RNA polymerase)-dependent plastid gene expression, very likely because of FTR connection with TRX z [98]. Besides the redox regulation of carbon metabolism enzymes, other important functions of TRXs in chloroplasts are their participation to stress response by regenerating thiol-dependent peroxidases and methionine sulfoxide reductases [99] and to the chlorophyll metabolism by regulating several enzymes of the tetrapyrrole biosynthesis pathway [100].

#### Beyond tetrapyrrole: chlorophyll and phytochromobilin

Interestingly, several enzymes of the chlorophyll metabolism are FDX targets and/or possess Fe–S clusters. The 7-hydroxymethyl chlorophyll a reductase (HCAR) is an enzyme binding two [Fe_4_S_4_] clusters and an FAD [101]. Besides there are four non-heme oxygenases, namely, pheophorbide *a* oxygenase (PAO), chlorophyll *a* oxygenase (CAO), translocon at the inner envelope membrane of chloroplasts 55 (TIC55), and protochlorophyllide (pchlide)-dependent translocon component of 52 kDa (PTC52) [102]. All possess a Rieske-type [Fe_2_S_2_] cluster and a mononuclear iron-binding domain. While the five enzymes are dependent on FDX, HCAR, CAO, and PTC52 are involved in chlorophyll synthesis, whereas PAO and TIC55 operate in its degradation. More precisely, CAO and HCAR are part of the chlorophyll cycle, the process of interconversion between chlorophyll *a* and chlorophyll *b*. The balance between chlorophyll *a*/*b* is important for both the stabilization and turnover of chlorophyll in the light-harvesting complexes (LHCs) in diverse physiological situations, notably during greening and senescence when LHCII is massively synthesized or degraded. CAO is a thylakoid membrane-anchored enzyme catalyzing the two steps of chlorophyll *a*-to-chlorophyll *b* oxidation [103]. PTC52 would catalyze an analogous oxidation, but using protochlorophyllide *a* as substrate. However, PTC52 is localized at the envelope [104], suggesting that it may have another dispensable function, being part of a translocation complex for the import of the protochlorophyllide oxidoreductase A (PORA) precursor in plastids [105]. Indeed, *A. thaliana ptc52* knock-out lines have a growth indistinguishable from wild-type plants (Table 1) [106]. On the contrary, an *Arabidopsis* mutant for *CAO*, named *chlorina1*, exhibits a pale green phenotype characterized by a chlorophyll *b* decrease [103] and is extremely sensitive to photooxidation due to the lack of chlorophyll–protein antenna complexes in PSII and to an increased production of singlet oxygen [107]. HCAR catalyzes the second half-reaction in chlorophyll *b*-to-chlorophyll *a* conversion, the first one being catalyzed by chlorophyll *b* reductases (CBR) [101]. While *hcar* mutants have no phenotype under standard growth conditions, they exhibit a stay-green phenotype after transfer to darkness (Table 1) [108].

In higher plants, chlorophyll is broken down to colourless linear tetrapyrroles in a series of reactions. One of these steps, the porphyrin ring opening of pheophorbide a, is catalyzed by PAO. This step occurs in senescent leaves and fruits, and requires FDXs and NADPH [109–111]. The PAO proteins possess a C-terminal transmembrane domain for their binding to the thylakoid membrane [104]. The *PAO* gene was identified by genetic studies and was initially referred to as accelerated cell death 1 (*ACD1*) in *Arabidopsis* [112] or *LLS1* (Lethal leaf spot 1) in maize [113]. Extinction of *PAO* in knock-out mutants or in antisense lines from different plant species leads to a light-dependent premature cell death phenotype, most likely due to cytotoxic effects of the increased pheophorbide *a* [109, 112, 114]. Similar to *hcar* mutants, *pao* mutants have a stay-green phenotype in dark [109, 114]. The product of the reaction catalyzed by PAO is red chlorophyll catabolite, which is then reduced by a FDX-dependent red chlorophyll catabolite reductase (RCCR) to yield the primary fluorescent chlorophyll catabolite (FCC), pFCC [115]. From this primary phyllobilin, a large variety of other phyllobilins is formed subsequently. Although a *tic55* mutant in *Arabidopsis* does not show any detectable phenotype, TIC55, which is localized in the inner membrane of the chloroplast envelope, is responsible for phyllobilin hydroxylation during senescence [104, 106]. This would be the last step in this subcellular compartment, and in that sense, TIC55 may contribute to chlorophyll catabolite export from plastids for their subsequent vacuolar detoxification.

A closely related molecule to chlorophyll and incidentally heme is phytochromobilin (PfB), the chromophore usually covalently bound to phytochromes of higher plants. All these molecules branch from protoporphyrin IX in the tetrapyrrole synthesis pathway. From the closed tetrapyrrole ring of heme, a heme oxygenase catalyzes the oxidative opening of this chain to yield biliverdin IXa. This molecule is then reduced into phytochromobilin by a PfB synthase. In higher plants, both types of enzymes are soluble and depend on FDXs for their activity [116, 117]. While PfB synthase is encoded by a single gene (*HY2*), the heme oxygenase is encoded by four members in *A. thaliana*, *HY1/HO1,* and *HO2*-*4* [118].

#### Macronutrient assimilation: similarities in nitrogen and sulfur assimilation pathways

The reductive assimilations of nitrogen and sulfur constitute two other chloroplastic metabolic processes, which rely on FDX-dependent Fe–S proteins. As already presented, sulfate assimilation is extremely important, because it provides cysteine, which is the source of sulfur for many molecules but also the substrate of cysteine desulfurases and a protein ligand in all plastidial Fe–S proteins known so far. Of the four enzymes/complexes, which allow forming cysteine from sulfate, two possess a Fe–S cluster. The second reaction, e.g, the transformation of adenosine 5′ phosphosulfate (APS) to sulfite, is catalyzed by adenosine 5′-phosphosulfate reductases (APR). There are three isoforms in *A. thaliana*, APR1-3, all localized in plastids. The enzymes are formed by two domains, a reductase domain, that bears a [Fe_4_S_4_] cluster, and a GRX domain at the C-terminus, that makes these enzymes glutathione-dependent [10]. The SIR catalyzes the next step, the six electron reduction of sulfite to sulfide. This FDX-dependent enzyme incorporates a siroheme, e.g, a heme whose iron atom is liganded by the thiolate ligand of a [Fe_4_S_4_] cluster, which is crucial for its activity. There is a single, essential, *SIR* gene in *Arabidopsis* and the protein is found exclusively in plastids. A weak allele mutant with about 25% SIR activity is viable but has a strongly retarded growth, pointing to the extreme importance of this enzyme for plant development [119].

The assimilation of inorganic nitrogen (mostly in the form of nitrate and ammonium) is another essential process for plants taking place in part in plastids. Nitrate will be reduced in two steps. The first one, catalyzed by nitrate reductase, gives nitrite, which is reduced to ammonia by a FDX-dependent nitrite reductase (NIR). As the SIR enzyme, NIR binds a siroheme that is mandatory for the six electron reduction of nitrite. This gene is also essential, since a mutant in barley does not grow in the absence of an external nitrogen source [120]. In the next steps, ammonia, including the part coming from the photorespiration process, is assimilated via glutamine synthetase (GS) which catalyzes the condensation of glutamate and ammonia into glutamine and via glutamate synthase (GOGAT) which forms two molecules of glutamate from glutamine and 2-oxoglutarate. There is evidence that NIR, GS, and GOGAT can form a complex within the chloroplast [121]. Plants possess two forms of chloroplastic GOGAT, which are dependent either on NADH or on FDX. All contain an FMN and a [Fe_3_S_4_] cluster. In *A. thaliana*, NADH-GOGAT is encoded by a unique gene (*GLT1*), whereas two genes encode Fd-GOGAT (*GLU1* and *GLU2*), GLU1 is the predominant form in leaves [122]. *Arabidopsis* mutants for *GLU2* and *GLT1* have no growth phenotype, although a decrease in the chlorophyll content was measured in the *glt1* mutant [123, 124]. An *Arabidopsis* mutant for *GLU1* has a respiratory phenotype, i.e, a dwarf and chlorotic phenotype in air which is no longer visible under high CO_2_ conditions [122, 123, 125]. Of importance for these pathways, it is worth mentioning that sirohydrochlorin ferrochelatase (SIRB), the enzyme responsible for the last step of siroheme biosynthesis by inserting ferrous iron into the tetrapyrrole ring of sirohydrochlorin, is a [Fe_2_S_2_] enzyme unlike bacterial orthologs. In this essential protein, the Fe–S cluster is not mandatory for the enzymatic reaction, but it might have a regulatory role [126].

#### Fatty acid biosynthesis

The biosynthesis of fatty acids is another crucial pathway occurring in plastids, which depends directly or indirectly on Fe–S proteins. First, the acetyl-coenzyme A, that is used as a building block for fatty acids, is generated by the plastidial pyruvate dehydrogenase (PDH) complex, its E2 subunit being lipoylated and thus dependent on the Fe–S containing lipoate synthase (see below). After the synthesis of saturated fatty acids, their conversion to unsaturated forms, which are required for membrane fluidity, is catalyzed by fatty acid desaturases. Some of them contain a di-iron center and are FDX-dependent proteins [127]. The FAB2 protein is a soluble stearoyl-ACP desaturase introducing the first double bond into stearoyl-ACP between carbons 9 and 10 to produce oleoyl-ACP (18:1 Delta9-ACP). The FAD5 protein attached to the chloroplast envelope inner membrane catalyzes the earliest step of 16:0 desaturation initiating the very rapid 16:0–16:1–16:2–16:3 desaturation of monogalactosyldiacylglycerol (MGDG), one of the four main classes of glycerolipids found in the photosynthetic membranes of higher plant chloroplasts with the digalactosyldiacylglycerol (DGDG), the phospholipid phosphatidylglycerol (PG), and the sulfolipid sulfoquinovosyldiacylglycerol (SQDG) [128]. Other plastidial linoleate/oleate desaturases (FAD4, 6, 7, 8) and the numerous FAD5-like proteins may also be dependent on FDX as they also probably contain di-iron centers.

#### Other metabolic processes

Additional FDX-dependent proteins are present in chloroplasts. Besides PAO, CAO, PTC52, and TIC55, the fifth non-heme oxygenase found in plants [102] is referred to as choline monooxygenase (CMO), because it was found to catalyze the oxidation of choline, the first step of glycine betaine biosynthesis in spinach [129]. However, this might not be the sole or main function, since *Arabidopsis* does not produce glycine betaine and expression of the *Arabidopsis* CMO-like gene in *E. coli* does not promote betaine synthesis [130]. This protein is unique to eukaryotic photosynthetic organisms as it is not found in cyanobacteria, supporting a recent evolution of this enzyme. No *Arabidopsis* mutant has been characterized so far, but antisense *CMO* transgenic sugar beet plants are susceptible to salt stress [131].

To conclude on this part, it is important to note that most of these enzymes are also expressed in plastids of non-photosynthetic tissues. In this context, FDXs are maintained reduced by FNR and NADPH generated in the oxidative pentose phosphate pathway, the reverse reaction compared to photosynthetic organs. A few other enzymes such as (1-hydroxy-2-methyl-2-(*E*)-butenyl 4-diphosphate synthase (HDS), zeaxanthine epoxidase, and β-carotene 3 hydroxylase 1, 2) have been also described as FDX-dependent proteins, but they will be discussed in the next sections. However, several additional proteins or pathways are yet unidentified. It is for instance worth mentioning that studies devoted to the isolation of FDX partners by proteomic approaches led to the identification of novel putative targets at least in cyanobacteria and *Chlamydomonas* [132, 133]. In this respect, a pyruvate:ferredoxin oxidoreductase (PFO), found in many unicellular eukaryotes, decarboxylates pyruvate to acetyl-coenzyme at the expense of FDXs [134]. The *C. reinhardtii* PFO possesses three distinct [Fe_4_S_4_] clusters. It may also contribute to the light-independent H_2_ production by passing electron to the hydrogenase [135].

### Biosynthesis of lipoic acid and thiamin cofactors and their dependent pathways

#### Requirement of two atypical radical-SAM enzymes

Beyond their role in electron transfer, Fe–S clusters are also important for enzyme catalysis, especially during the biosynthesis of vitamin B1/thiamin and of lipoic acid. Whereas thiamin is only synthesized in chloroplasts a lipoic acid biosynthesis pathway is present in both plastids and mitochondria. This is consistent with the existence of two distinct genes encoding a mitochondrial (mLIP) and a chloroplastic (cLIP) lipoate synthase [136].

Lipoic acid is synthesized from octanoic acid and thus via the fatty acid biosynthesis pathway by the addition of two sulfur atoms into the octanoyl group bound to an acyl carrier protein (ACP) via a radical-SAM mechanism. This reaction is catalyzed by lipoic acid synthase [136]. It is important to note that lipoic acid is synthesized attached to proteins and no free lipoic acid is produced. The *E. coli* Lip5 binds two [Fe_4_S_4_] clusters [137]. One cluster, coordinated by cysteines present in a Cx_3_Cx_2_C motif common to all classical radical-SAM enzymes, is required for the formation of the activated adenosyl radical from SAM molecules. The second cluster, coordinated by a Cx_4_Cx_5_C motif specific of lipoic acid synthases, was suggested to provide the sulfur atoms and thus to be degraded at each turnover of the enzyme. The presence of a Fe–S cluster has not yet been demonstrated in plant cLIP, but *Arabidopsis* cLIP possesses both cysteine motifs and is able to complement the *E. coli lip5* mutant [136]. Plants impaired in *cLIP* have not been characterized yet, but *Arabidopsis* mutants for genes involved in the synthesis of lipoic acid in mitochondria are lethal [138].

Thiamin is made of pyrimidine and thiazole heterocycles, both being synthesized in the chloroplast. The synthesis of the thiazole moiety involves a 4-methyl-5-*b*-hydroxyethylthiazole phosphate (HET-P) synthase (THI1) forming an adenylated thiazole intermediate (ADT) at the expense of nicotinamide adenine dinucleotide (NAD) and glycine, ADT, which is then hydrolyzed to HET-P [139]. The pyrimidine heterocycle is derived from purine biosynthesis. The first step in the synthesis of the pyrimidine moiety is catalyzed by the 4-amino-2-methyl-5-hydroxymethylpyrimidine phosphate (HMP-P) synthase (THIC), a radical-SAM Fe–S enzyme that forms HMP-P from 5-aminoimidazole ribonucleotide (AIR) and SAM. In contrast to canonical radical-SAM enzymes, all THIC proteins harbour a Cx_2_Cx_4_C motif involved in the binding of a [Fe_4_S_4_] cluster in their C-terminal part [140, 141]. Then, an HMP-P kinase/thiamin monophosphate (ThMP) pyrophosphorylase (TH1) phosphorylates HMP-P to HMP-PP but also condenses the latter compound to HET-P to form ThMP. This ThMP is transformed into the diphosphate form ThDP in the cytosol through the action of two consecutive enzymes before being redistributed to mitochondria and plastids. THIC is encoded by a single essential gene in *Arabidopsis* [140]. An *Arabidopsis thic* mutant is lethal at the cotyledon stage unless supplemented with thiamin [140, 142]. Another family of plastidial Fe–S enzymes is linked indirectly to thiamin biosynthesis, because they catalyze the first committed step of the de novo synthesis of purine in chloroplasts. The glutamine phosporibosyl pyrophosphate amidotransferases (ATases, also known as GPAT) catalyze the amination of 5-phosphoribosyl-1-pyrophosphate (PRPP) to 5-phospho-ribosylamine (PRA) with the concomitant conversion of glutamine into glutamate [143]. After four additional steps, PRA is transformed into AIR, the THIC substrate. In *Arabidopsis*, ATase is encoded by a family of three genes (*ATase1* to *ATase3*) which are expressed in various tissues at different levels [144, 145]. Whereas *E. coli* ATase does not require a Fe–S cluster as cofactor, the human enzyme uses a [Fe_4_S_4_] cluster. Based on the conservation of the involved cysteines, the three *A. thaliana* isoforms should also bind a Fe–S cluster. Whereas *Arabidopsis ATase1* mutant has no growth phenotype mutants lacking *ATase2* exhibit strong growth retardation with bleached leaves (Table 1) [144]. In the latter mutant exhibiting a decreased capacity in chloroplast protein import, cells are smaller in size [144, 146].

#### A single lipoic acid-dependent enzyme but several thiamin-dependent enzymes in plastids

In plastids, the only known lipoic acid-dependent enzyme is PDH. A similar complex is found in plant mitochondria, but it uses lipoic acid synthesized in this compartment, as does another citric acid cycle enzyme, the α-ketoglutarate dehydrogenase or 2-oxoglutarate dehydrogenase complex, but also two complexes involved in the amino acid metabolism, the glycine cleavage complex, and the branched-chain oxoacid dehydrogenase (BCDH) complex. On the other hand, there is a single pathway for the synthesis of ThDP which is used as a coenzyme by many enzymes of the primary metabolism, notably involved in the catabolism of sugars and amino acids, and found in the chloroplasts, mitochondria, and cytosol [139]. In plastids, besides the PDH complex, thiamin is also a cofactor for transketolase (TK) of both the Calvin–Benson cycle and the non-oxidative pentose phosphate pathway, for 1-deoxy-d-xylulose 5-phosphate synthase (DXS) of the methylerythritol phosphate (MEP) pathway and for acetohydroxy acid synthase (AHAS) of the branched-chain amino acid (BCAA) biosynthesis pathway [139].

##### The central pyruvate dehydrogenase complex

PDH catalyzes the decarboxylation of pyruvate into acetyl-coA that is used in particular for fatty acid synthesis as already mentioned [128]. It consists of three subunits, E1–E3, each requiring a different cofactor. Thiamin is bound to the pyruvate dehydrogenase subunit (E1), whereas the lipoic acid is covalently attached to the dihydrolipoyl acyltransferase subunit (E2) and an FAD is bound to the dihydrolipoamide dehydrogenase subunit (E3). The attached lipoyl moiety functions as a carrier of reaction intermediates among the active sites of the components of the complex. The E3 subunit has a key regulatory role by reoxidizing the lipoamide cofactor and thus completing the catalytic cycle. The disruption of the gene encoding the E2 subunit of plastidial PDH results in an early embryo-lethal phenotype in *Arabidopsis* [147].

##### Branched-chain amino acid biosynthesis

The AHAS protein is involved in the first steps of BCAA biosynthesis. This is a heterodimer, composed of separate catalytic and regulatory subunits, which catalyzes the conversion of two molecules of pyruvate into 2-acetolactate used for valine and leucine synthesis or of one molecule of pyruvate and one molecule of 2-oxobutanoate into 2-aceto-2-hydroxybutyrate used for isoleucine synthesis [148]. Interestingly, two Fe–S enzymes named dihydroxyacid dehydratase (DHAD) and isopropylmalate isomerase (IPMI) are also required for BCAA synthesis. DHAD catalyzes the penultimate step before the formation of isoleucine and valine, e.g., the dehydration of 2,3-dihydroxy-3-isovalerate or 2,3-dihydroxy-3-methylvalerate to the 2-oxo acids (3-methyl-2-oxobutanoate or 3-methyl-2-oxopentanoate). In *Arabidopsis*, there is a single essential gene for DHAD [149]. However, *Arabidopsis* mutants with intermediate DHAD levels obtained by an RNAi approach indeed have reduced amounts of BCAA in roots, which cause a short root phenotype [149]. The only biochemical characterization performed so far has been done with an enzyme purified from spinach leaves. Unlike the *E. coli* enzyme, which incorporates a [Fe_4_S_4_] center, the spinach enzyme incorporates a [Fe_2_S_2_] cluster required for activity [150]. Leucine biosynthesis requires an additional Fe–S enzyme for the late reactions. The isopropylmalate isomerase catalyzes the reversible conversion of 2-isopropylmalate into 3-isopropylmalate. In plants, IPMI consists of a heterodimer composed of a large (LSU) and a small (SSU) subunit encoded by one and three genes in *A. thaliana,* respectively [151]. The genetic analyses demonstrated that *A. thaliana* knock-down mutants for the large subunit, which binds a [Fe_4_S_4_] center, display a severe delay in development [151, 152]. Concerning small subunits, the SSU1 protein is required for viability, unlike SSU2 and SSU3, which might be redundant, because *A. thaliana* mutants have no phenotype [151, 153]. In addition to a role in leucine biosynthesis, IPMI is involved in the biosynthesis of glucosinolates, sulfur-containing secondary metabolites, serving for defence reactions. This is consistent with the fact that an identical reaction type exists for the Met chain elongation cycle for glucosinolate formation and that *A. thaliana* mutant plants for the large subunit accumulate both Leu biosynthesis and Met chain elongation intermediates [151].

##### Isoprenoid biosynthesis and its derived molecules

Isoprenoids are very diverse metabolites, central to plant development. We have already discussed the biosynthesis of chlorophylls, which consist of a tetrapyrrole ring with an attached isoprenoid-derived phytol chain, but many other isoprenoids are present in plastids such as α-tocopherol, phylloquinone, plastoquinone, and carotenoids to cite only the most important. Moreover, several plant hormones are derived from carotenoids. All isoprenoids are derived from a prenyl diphosphate (prenyl-PP) precursor, which is synthesized by two independent pathways, the cytosolic mevalonate (MVA) pathway, and the plastidial 2-*C*-methyl-d-erythritol 4-phosphate (MEP) pathway [154]. The latter pathway is dependent on both Fe–S and thiamin (TK and DXS)-dependent enzymes. In *E. coli*, the Fe–S proteins belonging to this MEP pathway are the only one that are completely essential. Indeed, the lethality of *sufa*-*isca* and *erpa* mutants observed under aerobiosis is suppressed by expressing the eukaryotic mevalonate pathway, which does not rely on Fe–S proteins [54, 155].

Besides its involvement in the Calvin–Benson cycle where it catalyzes the formation of ribose-5-phosphate and xylulose-5-phosphate from sedoheptulose-7-phosphate and glyceraldehyde-3-phosphate (GA-3P), TK operates in the opposite direction in the non-oxidative pentose phosphate pathway, forming GA-3P, which is then condensed to pyruvate to form 1-deoxy-d-xylulose 5-phosphate (DXP), a reaction catalyzed by DXP synthase (DXS). Although poorly characterized in plants, it was demonstrated that antisense tobacco plants with variable TK levels have a marked shoot weight decrease [156]. For the most affected lines, a decrease in chlorophylls and carotenoids was measured which is consistent with the importance of DXP for the MEP pathway. Surprisingly, overexpression of an *A. thaliana* chloroplastic TK in tobacco leads to chlorosis, which is annihilated by thiamin supplementation [157]. In *A. thaliana*, *DXS* is an essential gene [158]. In sense and antisense *Arabidopsis* lines exhibiting altered levels of DXS, both the chlorophylls, carotenoids, tocopherols, gibberellin, and abscisic acid contents and the growth and germination rate are slightly affected [159]. Finally, downstream of DXS, two Fe–S proteins are required to form two key intermediates in isoprenoid biosynthesis: isopentenyl diphosphate (IPP) and dimethylallyl diphosphate (DMAPP). Both the 1-hydroxy-2-methyl-2-(*E*)-butenyl 4-diphosphate synthase (HDS/ISPG) and the 1-hydroxy-2-methyl-2-(*E*)-butenyl 4-diphosphate reductase (HDR/ISPH/LytB) bind a [Fe_4_S_4_] cluster [160, 161], FDX being able to provide electrons to HDS [162]. Plant mutants disrupted in *ISPG* or *ISPH* gene have a severely impaired chloroplastic development that causes an albino phenotype [163–165].

At least two enzymes participating in the carotenoid biosynthesis pathways are dependent on FDXs. Without describing all the steps, the β-carotene 3 hydroxylase 1, 2 which contains a di-iron center catalyzes two successive steps, the transformation of all-trans β-carotene to β-cryptoxanthin and then to zeaxanthin. Then, the flavoprotein zeaxanthin epoxidase catalyzes the conversion of zeaxanthin to antheraxanthin and then to violaxanthin [166]. These four steps require oxygen and FDX as an electron donor. Other proteins in this pathway might, in fact, be dependent on FDXs. For instance, there are several cytochrome P450 monooxygenases participating in this pathway (and other pathways) in plastids, whose electron donors/acceptors are yet unknown.

Derived from carotenoids, strigolactones (SL) are plant hormones having diverse functions in plant growth and development. Their biosynthesis begins with the conversion of all-trans β-carotene to 9-*cis*-β-carotene, a reaction performed by a β-carotene isomerase named DWARF27. This protein, found from algae to higher plants, and first characterized in rice is a Fe–S enzyme [167]. The *Arabidopsis* genome encodes three orthologs. An *Arabidopsis* mutant for one of these genes and a rice mutant have shoot branching phenotypes, but it remains relatively weak compared to other mutants affected in SL biosynthesis [168].

### Are there other plastidial Fe–S proteins to discover?

Some Fe–S proteins, such as NEET, have been recently identified or characterized in plants. Unlike mitoNEET, which is bound to the outer membrane of mitochondria in animals owing to a membrane anchoring extension, the *Arabidopsis* NEET protein is located exclusively in the chloroplast stroma [169]. As its vertebrate counterparts, *Arabidopsis* NEET forms dimers; each monomer harbouring an atypical [Fe_2_S_2_] cluster coordinated by three Cys and one His [170]. While obtaining knock-out plants may have been hampered by the fact, it is an essential gene, *Arabidopsis* lines with reduced *AtNEET* transcript levels exhibit late greening, delayed bolting, and early senescence. Moreover, these plants accumulate ROS and have an altered sensitivity to Fe levels, which led to the proposal that AtNEET likely plays a role in the regulation of Fe homeostasis [170]. From its capacity to transfer its Fe–S cluster to a FDX in vitro [170], it may be hypothesized that NEET could be part of the SUF machinery and facilitate the trafficking of Fe–S clusters towards certain client proteins.

Another reason why we expect to discover novel Fe–S proteins is that some proteins may be specific to photosynthetic organisms because of their atypical structure organization or their involvement in specific plastidial functions. An interesting example in this regard is SUFE3, a chimeric protein formed by an SUFE domain fused to a quinolinate synthase domain, NADA [18]. This enzyme, which carries a [Fe_4_S_4_] cluster indispensable for its activity and thus crucial for NAD biosynthesis, is the sole NADA representative of *A. thaliana*. The fact that the Fe–S cluster in SUFE3 can be reconstituted using its own SUFE domain in the presence of NFS2, cysteine, and ferrous iron may render this protein independent on the SUFBCD scaffold complex [18].

Other Fe–S protein-dependent processes likely remain to be identified in plastids as in other subcellular compartments. For instance, the affinity purification strategy used for cyanobacterial and algal enzymes indicates that numerous FDX-dependent processes await identification [132, 133]. The same is true in mitochondria where the roles and partners of the two FDXs are unknown. On the other hand, novel Fe–S proteins will be undoubtedly identified in the future thanks to the dozens of annotated sequenced genomes now available for model plants and to the ever larger collections of available *Arabidopsis* mutants. The reasons why it is not trivial to isolate them is that the sensitivity of these metallic cofactors to oxygen may hamper the isolation of holoproteins and predictions of Fe–S proteins from the protein primary sequences are often impossible, because there is no universal signature for identifying Fe–S cluster ligands.
