# Correction to: Roles and maturation of iron–sulfur proteins in plastids

**DOI:** 10.1007/s00775-018-1570-8

**Published:** 2018-05-29

**Authors:** Jonathan Przybyla-Toscano, Mélanie Roland, Frédéric Gaymard, Jérémy Couturier, Nicolas Rouhier

**Affiliations:** 1 Université de Lorraine, Interactions Arbres-Microorganismes, UMR1136, 54500 Vandoeuvre-lès-Nancy, France; 2 Biochimie et Physiologie Moléculaire des Plantes, CNRS/INRA/Université Montpellier 2, SupAgro Campus, 34060 Montpellier, France

## Correction to: JBIC Journal of Biological Inorganic Chemistry 10.1007/s00775-018-1532-1

The article “Roles and maturation of iron–sulfur proteins in plastids”, written by Jonathan Przybyla‑Toscano, Mélanie Roland, Frédéric Gaymard, Jérémy Couturier, Nicolas Rouhier was originally published electronically on the publisher’s internet portal (currently SpringerLink) without open access.

The copyright of the article changed on 25, May to © The Author(s) 2018 and the article is forthwith distributed under the terms of the Creative Commons Attribution 4.0 International License (http://creativecommons.org/licenses/by/4.0/), which permits use, duplication, adaptation, distribution and reproduction in any medium or format, as long as you give appropriate credit to the original author(s) and the source, provide a link to the Creative Commons license and indicate if changes were made.

The original article has been corrected.

